# RNA sequencing of intestinal mucosa reveals novel pathways functionally linked to celiac disease pathogenesis

**DOI:** 10.1371/journal.pone.0215132

**Published:** 2019-04-18

**Authors:** Maureen M. Leonard, Yu Bai, Gloria Serena, Kourtney P. Nickerson, Stephanie Camhi, Craig Sturgeon, Shu Yan, Maria R. Fiorentino, Aubrey Katz, Barbara Nath, James Richter, Matthew Sleeman, Cagan Gurer, Alessio Fasano

**Affiliations:** 1 Mass General Hospital *for* Children and Division of Pediatric Gastroenterology and Nutrition, Harvard Medical School, Boston, Massachusetts, United States of America; 2 Center for Celiac Research and Treatment, Mucosal Immunology and Biology Research Center, Boston, Massachusetts, United States of America; 3 Celiac Research Program, Harvard Medical School, Boston, Massachusetts, United States of America; 4 Regeneron Pharmaceuticals, Tarrytown, New York, United States of America; 5 Graduate Program in Life Sciences, University of Maryland, Baltimore, Maryland, United States of America; 6 Department of Gastroenterology, Massachusetts General Hospital, Boston, Massachusetts, United States of America; Tulane University, UNITED STATES

## Abstract

**Background & aims:**

The early steps in the pathophysiology of celiac disease (CD) leading to loss of tolerance to gluten are poorly described. Our aim was to use RNA sequencing of duodenal biopsies in patients with active CD, CD in remission, and non-CD controls to gain insight into CD pathophysiology, identify additional genetic signatures linked to CD, and possibly uncover targets for future therapeutic agents.

**Methods:**

We performed whole transcriptome shotgun sequencing of intestinal biopsies in subjects with active and remission CD and non-CD controls. We also performed functional pathway analysis of differentially expressed genes to identify statistically significant pathways that are up or down regulated in subjects with active CD compared to remission CD.

**Results:**

We identified the upregulation of novel genes including IL12R, ITGAM and IGSF4 involved in the immune response machinery and cell adhesion process in the mucosa of subjects with active CD compared to those in remission. We identified a unique signature of genes, related to innate immunity, perturbed exclusively in CD irrespective of disease status. Finally, we highlight novel pathways of interest that may contribute to the early steps of CD pathogenesis and its comorbidities such as the spliceosome, pathways related to the innate immune response, and pathways related to autoimmunity.

**Conclusions:**

Our study confirmed previous findings based on GWAS and immunological studies pertinent to CD pathogenesis and describes novel genes and pathways that with further validation may be found to contribute to the early steps in the pathogenesis of CD, ongoing inflammation, and comorbidities associated with CD.

## Introduction

Celiac disease (CD) is a chronic systemic autoimmune disease that occurs globally in genetically predisposed individuals in response to ingestion of gluten-containing grains[1]. While the pathognomonic damage occurs in the small intestine, clinical manifestations are varied and include both intestinal and extra-intestinal symptoms[2]. CD is a unique autoimmune disease in that there is a strong genetic component, notably human leukocyte antigen (HLA) DQ2 and DQ8[3]. During active disease, the production of autoantibodies against tissue transglutaminase (anti-tTG2) can be measured in the blood to indicate the presence of disease. While HLA DQ2 and/or DQ8 and gluten ingestion are necessary to develop CD, they are not sufficient as most individuals with compatible HLA do not develop CD. Furthermore, CD can develop at any age[4] but what leads ultimately to this loss of tolerance to gluten and development of intestinal inflammation is unknown.

Despite the well described pathogenic role of the adaptive immune response in CD, a complete understanding of the pathophysiology of CD, particularly concerning the early steps leading to loss of tolerance to gluten, are poorly defined[2]. Additionally, biomarkers confirming mucosal recovery for patients with CD on a gluten free diet are lacking. For this reason, we sought to use whole transcriptome shotgun sequencing of duodenal biopsies for an analysis to gain more insight on CD pathophysiology and possibly identify biomarkers suggestive of active disease and mucosal recovery, including pathways which may lead to the discovery of future targets for therapeutic interventions. Our work provides a base from which further investigation may validate and mechanistically link these findings to previously unknown aspects of the pathogenesis of CD.

More than 57 non-HLA associated genetic loci contributing to CD have been identified, which together with the HLA contribution, explains less than 50% of CD heritability[5–7]. Molecular analysis of the pathogenic mechanisms in CD have assessed gene expression profiles using small intestinal biopsies and specific cell types[8–15]. Previous work has utilized RNA sequencing of whole duodenal biopsies to investigate expression of genes associated with tight junctions in patients with active CD, remission CD, and control patients[15]. In this study, we performed whole transcriptome shotgun sequencing in duodenal biopsies in subjects with active CD compared to those with CD in remission and compared these subjects to individuals with CD-compatible HLA genotypes who are not affected by CD. This approach allowed us to gain insight regarding specific pathways possibly involved in the cascade of events upstream to the adaptive immune response that contribute to the loss of tolerance to gluten. Furthermore, through a focus on molecular signatures that are conserved in patients with CD regardless of the disease activity and those signatures that differentiate active CD from CD in remission, we shed insight on constitutively-involved pathways that may be at play not only in CD but also in other chronic inflammatory diseases.

## Results

### Comparison of gene expression in patients with active CD, CD in remission, and non-CD control patients

**Fig 1:**
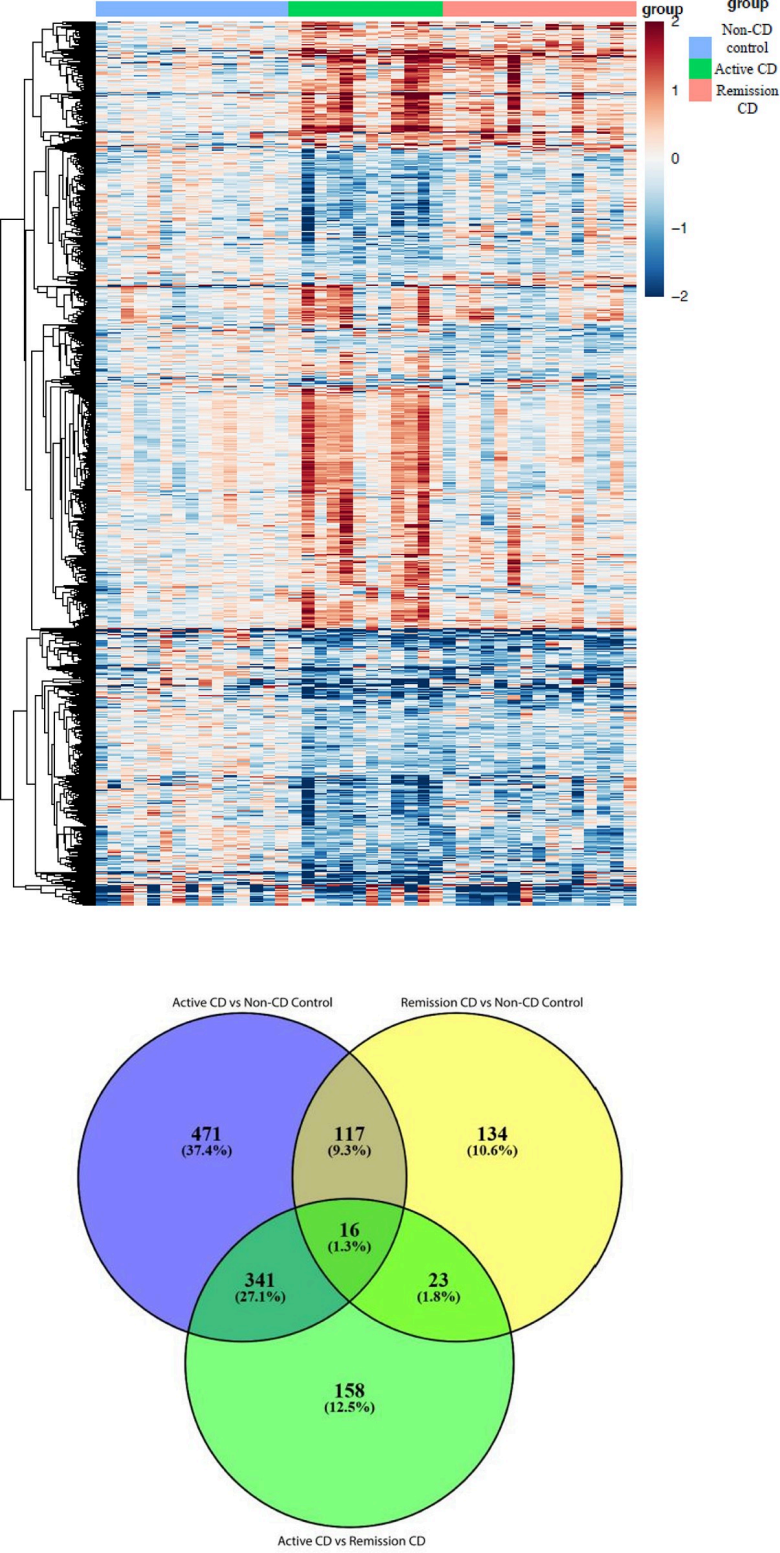
Comparison of gene expression in non-cd control, active CD, and remission CD subjects. Heatmap shows non-CD control subjects (blue), active CD subjects (green) and remission CD subjects (pink) with each subject’s data represented vertically. The color scale show gene expression with dark blue indicating downregulation, light blue indicating decreased expression, light red indicating increased expression, and red indicating upregulation. (A)Gene expression in non-CD control, active CD, and remission CD subjects is notable for the distinct differences between each group. (B) A venn diagram of unique and overlapping gene signatures shows genes exclusively altered in active CD (purple) (471) and remission CD (yellow) (134). An additional 117 genes are constituently changed in subjects with CD irrespective of the disease status but not non-CD controls.

A heatmap comparing the three study groups shows clear gene expression differences (Fig 1A). The profound differences between groups in this analysis is testimonial of the strict adherence to subject enrollment criteria, quality of sample collection, and analysis. Our analysis identified 945 genes significantly different when comparing subjects with active CD to non-CD control subjects (S1 Fig). We identified 290 genes significantly different between subjects with CD in remission and non-CD control subjects. We identified 538 genes significantly different between subjects with active CD and CD in remission. Finally, 117 genes were common to subjects with active and remission CD (S2 Fig). A venn diagram (Fig 1B) shows the unique signature of genes perturbed exclusively in active CD (471) and those perturbed exclusively in remission CD (134). There are an additional 117 genes, mainly involved in innate immune response, that are changed constituently in subjects with CD irrespective of the disease status (active or remission). Due to the large number of genes differentially expressed between subjects with active CD and remission CD, we sought to further analyze these differentially expressed genes by functional pathway analysis.

To validate the RNAseq analysis, we performed reverse transcriptase (RT) real time polymerase chain reaction (PCR) for a subset of genes that showed significantly altered expression in active CD patients. Our data confirmed the RNA seq analysis with IFNg (p≤0.05), IL10 (p≤0.05), MCM2 (p≤0.005), CDC45 (p≤0.001) and CXCL11 (p≤0.05) significantly over-expressed in the active celiac populations as compared to control subjects. However, RT PCR for CXCR4, CADM1 and CLDN10 did not confirm RNA seq analysis (S3 Fig). We also performed immunohistochemistry on duodenal biopsies from healthy control patients, patients with active CD, and patients with CD in remission (S4 Fig). Our data confirmed the RNA seq analysis with MCM2 and CDC45 over-expressed in patients with active CD compared to patients with CD in remission and healthy control subjects. Please note that there is unspecific staining in the figure and CDC45 and MCM2 specific staining is seen in the crypt and crypt nuclei respectively.

### Pathway analysis in subjects with active CD, CD in remission, and non-CD control subjects

We used two approaches to analyze pathways associated with patients with active CD and CD in remission. First, to analyze the enriched pathways in the unique genetic signatures related to active CD, and in those signatures common to active CD and CD in remission, we applied the Pathway Enrichment module of NextBio as described in the methods[16]. These signatures were filtered based on the fold change and p-value thresholds followed by segmentations via the Venn Diagram, and thus were no longer genome-wide. Only specific types of enrichment tests including the running Fisher algorithm offered by NextBio were applicable. Other methods such as Gene Set Enrichment Analysis (GSEA) were not suitable for the non-genome-wide signature lists. Using this approach, we identified 169 significant pathways unique to subjects with active CD compared to CD subjects in remission or non-CD control subjects (S1 Table). The top perturbed pathways exclusively upregulated in patients with active CD were overwhelmingly related to the cell cycle which was noted in 6 of the top 10 pathways. Other highly significant pathways included PLK-1 signaling event, FOXM1 transcription factor network, retinol metabolism, and other drug metabolism.

We also identified 8 pathways that were constitutively changed in subjects with CD, irrespective of their disease state, that were distinct from non-CD control subjects. Intriguingly all pathways were upregulated and most of them, such as NK cell mediated cytotoxicity and chemokine signaling pathway, listed in Table 1, are related to the innate immune system. Other notably upregulated pathways, such as interferon signaling link the innate immune system to the adaptive immune system. Antigen processing and presentation, a well described key to CD pathogenesis is also upregulated [2]. These results suggest that individuals with CD have a constitutive upregulation of pathways related to innate immune surveillance.

**Table 1:** Pathways common to active and remission CD but not non-CD controls.

Biogroup Name	Genes(#)	Direction	P-Value
Natural killer cell mediated cytotoxicity	6	up	1.00E-08
Ras-Independent pathway in NK cell-mediated cytotoxicity	3	up	3.90E-08
Antigen processing and presentation	5	up	5.10E-08
Genes involved in Immunoregulatory interactions between a Lymphoid and a non-Lymphoid cell	4	up	9.30E-07
Graft-versus-host disease	3	up	1.30E-05
Genes involved in Interferon alpha/beta signaling	3	up	5.20E-05
Genes involved in Interferon Signaling	3	up	0.0007
Chemokine signaling pathway	3	up	0.001

**Fig 2:**
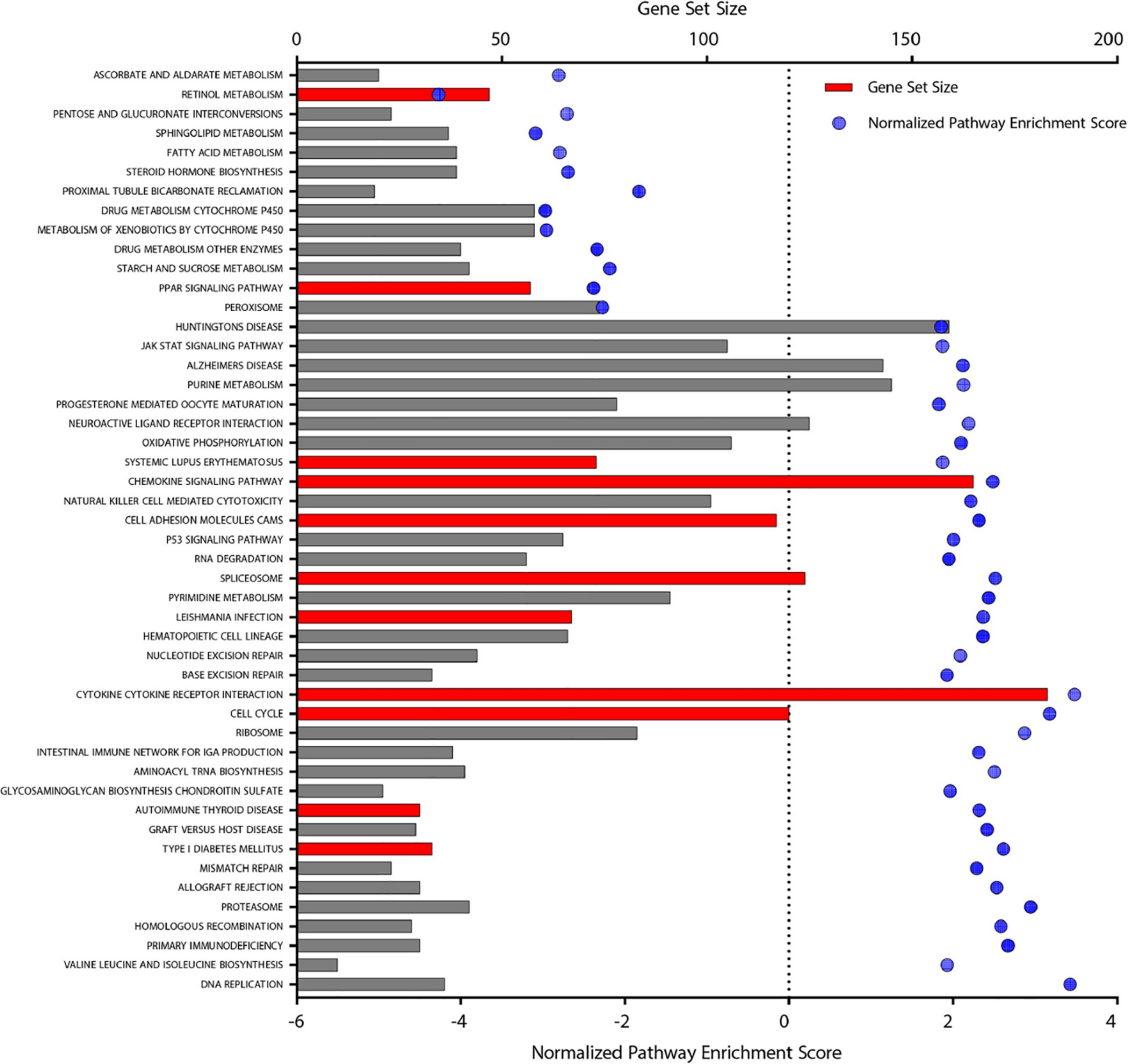
Significant pathways identified in active CD compared to remission CD subjects according to GSEA analysis using KEGG pathways. Pathways significantly altered in patients with active CD compared to remission CD per GSEA are shown horizontally. The gene set size is depicted by the size of the bar associated to each pathway per the vertical axis on the left. The normalized pathway enrichment score is shown on the right vertical axis and is depicted by the circles associated with each path. Pathways in red are discussed in detail.

We ultimately chose to focus on the pathways that were significantly different between subjects with active CD and those in remission. To do this we took a second approach to analyze pathways in an effort to minimize bias that may be introduced by using the empirically defined fold change and p-value cutoffs. Therefore, we chose to perform gene set enrichment analysis (GSEA) for the active vs. remission analysis as it does not require any pre-selection and utilizes the genome-wide signature list. In this way, all genes were considered in the comparison of active CD compared to remission CD for analysis. KEGG pathway database was used for the GSEA analysis. We identified 48 KEGG pathways of significant interest (Fig 2). Of these, 35 pathways were significantly upregulated and 13 pathways were significantly downregulated in subjects with active CD compared to CD subjects in remission (Fig 2). Highly enriched pathways that were upregulated included DNA replication, valine, leucine, isoleucine biosynthesis, primary immunodeficiency, homologous recombination, and proteasome. Alternatively, enriched pathways which were downregulated included ascorbate and aldarate metabolism, retinol metabolism, pentose and glucuronate interconversions, sphingolipid metabolism, and fatty acid metabolism. When ranked by significance, the top three pathways, all of which were upregulated, included: cytokine-cytokine receptor interaction, chemokine signaling pathway, and cell cycle. Our results that follow highlight the top three perturbed pathways significantly different between subjects with active CD compared to those in remission as well as other novel pathways of interest to us which may shed light on the early events leading to the development of CD.

### Immune response: Cytokine-cytokine receptor interaction and chemokine signaling pathway

Our results show the signaling pathways of cytokines (S5 Fig) and chemokines (S6 Fig) as two of the top three perturbed pathways upregulated in subjects with active CD compared to those in remission. The identification of these previously described pathways in the innate and adaptive immune systems highlights the strength and validity of our analytical approach [17].

Neutrophil recruitment in the intestinal lamina propria represents one of the earliest steps in the cascade of events in response to gluten [18, 19]. We found an upregulation of neutrophils chemo attractants and their regulators (IL-8, CXCL1, CXCL2, CXCL3, CXCL6 and CXCR4) in subjects with active CD compared to those in remission. Furthermore, our data also showed an increased expression of CXCR3 and its ligands (CXCL9, CXCL10, CXCL11). The interaction between gliadin and CXCR3 is known to trigger IL-8 secretion by epithelial and immune cells resulting in neutrophil migration [20, 21]. Our analysis confirmed that both EGF and IL-15 pathways were upregulated in subjects with active CD compared to those in remission and that IL-15 dependent macrophage-attracting chemokines (CCL20 and its receptor CCR6) were also upregulated. Similarly, upregulation of type -1 IFNs, IL1B, CCR2, CCR7 and CCR4 were found in active CD subjects supporting the previously described contribution of the innate immune component to the pathogenesis of the disease and, more specifically, the recruitment of macrophages and dendritic cells [22–25].

Our data further demonstrates the upregulation of Th1- and Th17-derived cytokine pathways such as IFNy, TNFa, IL-2, IL-10, IL-17, and IL-21 are consistent with previous work which highlights the role of the adaptive immune response and the autoimmune nature characteristic of the chronic inflammation in subjects with active CD.

We also identified novel genes involved in the immune response machinery, IL-12RB1and IL-12RB2, upregulated in the mucosa of patients with active CD which was consistent with our findings that IL-12 signaling was upregulated in subjects with active CD compared to those in remission.

### Cell cycle

Our analysis shows upregulation of CDX genes related to the MAPK signaling pathway in active CD compared to CD in remission. Additional findings were the upregulation of molecules related to DNA replication (MCM helicase and Cdc45) and of c/myc oncogene (S7 Fig) [26].

### Other pathways of interest

#### Cell adhesion molecules

The cell adhesion pathway analysis revealed changes in subjects with active CD compared to remission CD (S8 Fig). Specifically, the claudin (CLDN) family showed a down-regulation of the barrier forming CLDN 4,12, 15 and up-regulation of the pore forming CLDN 2 and 10. Barrier forming CLDN 1 and 5 were upregulated along with CLDN18. C1orf106, a gene shown to regulate epithelial adherens junction stability, was downregulated in subjects with active CD compared to subjects with CD in remission and non-CD control subjects again suggesting a role for increased intestinal permeability in subjects with active CD[27]. Additionally, we detected the up-regulation of IGSF4, a nectin-like molecule involved in a variety of cell-cell junctions, such as adherens junctions and tight junctions in epithelial cells [28].

Our analysis also revealed the upregulation of intercellular adhesion molecules (ICAMs), members of the immunoglobulin superfamily. Our analysis revealed upregulation of ICAM1-3, a molecule present on antigen presenting cells, both cytotoxic and helper T cells, and B cells. In addition, ITGAM, an adhesion molecule expressed primarily on monocytes and neutrophils, which is involved in activation, adherence, and migration of leukocytes has been found upregulated in samples from our active CD subjects.

#### Spliceosome

Spliceosome related pathways was one of the top 5 perturbed pathways in subjects with active CD compared to those in remission. Specifically, the expression of several genes coding for proteins necessary for the assembly (prp5), activation (prp2) and finally disassembly (prp22, prp43) of the spliceosome complex were significantly increased in the active CD patient group (S9 Fig)

#### IL-17, retinoic acid and associated autoimmune diseases

We found an upregulation in IL-17 signaling in subjects with active CD compared to subjects with CD in remission in agreement with previous work[29, 30]. Our data showed a down-regulation of the vitamin A/retinoic acid pathways suggesting a possible correlation between the Th17 mediated immune response that characterizes active CD and alterations in retinol metabolism (S10 Fig). Additionally, the pathways related to systemic lupus erythematosus (S11 Fig), type 1 diabetes (T1D) (S12 Fig), and autoimmune thyroiditis (S13 Fig), were significantly upregulated in subjects with active CD compared to those in remission.

## Discussion

Our analysis of subjects with active CD compared to those in remission and non-CD controls using whole transcriptome shotgun sequencing of duodenal biopsies revealed results consistent with many of the previously described pathways identified in the pathogenesis of CD, suggesting that our analysis is robust. Our finding that patients with CD, irrespective of disease status, have a constitutive upregulation of pathways related to innate immunity confirms the important role of the innate immune system in CD pathogenesis. By focusing on the statistically significant pathways that are up or down regulated in subjects with active CD compared to those in remission, we identified novel pathways of interest such as the spliceosome pathway that may contribute to the early steps involved in losing tolerance to gluten. We also identified novel genes involved in the immune response machinery (IL-12RB1and IL-12RB2) and cell adhesion process (IGSF4 and ITGAM) in the mucosa of subjects with active CD compared to those in remission. Together, our findings support a possible link between the microbiome, innate immune response, and the development of CD and highlight possible associations that with future validation may lead to crucial knowledge of the steps leading to loss of tolerance to gluten. Finally, we also suggest possible molecular mechanisms for the association with other autoimmune diseases and persistent enteropathy despite a strict gluten free diet. Our data serve to act as a launching point to suggest areas of further investigation and validation for CD pathogenesis, treatment, and associations with other autoimmune disease.

Our findings that patients with active CD had an upregulation in cytokine-cytokine receptor interaction and the chemokine signaling pathway is expected given our understanding of CD pathogenesis and the inflammation associated with active CD. Our data have also provided possible insights into genes involved in the immune response. While upregulation of IFNy in subjects with active CD is well described, our finding of IL-12 upregulation in the mucosa of subjects with active CD is novel. Previous work has identified elevated IL-12, specifically IL-12RB2, in the mucosa of patients with active Crohn’s disease [31]. We found upregulation of both IL-12RB1 and IL-12RB2 in subjects with active CD compared to those in remission. While the molecular basis of Th1 differentiation is not well described, STAT-4 and IRF-1 have been associated with IL-12 and IFNy signaling in T cells and involved in regulating cytokine production of Th1 cells at the transcriptional level. Our analysis found a significant upregulation of IRF-1 in subjects with active disease compared to controls.

Our analysis identified the upregulation of cell adhesion molecules such as IGSF4 and others involved in leukocyte transendothelial migration such as ITAGAM (S8 Fig) in active CD. ITGAM which combines with the beta 2 chain (ITGB2, also found upregulated in our analysis) to form a leukocyte-specific integrin referred to as macrophage receptor 1 [32] is important in the adherence of neutrophils and monocytes to stimulated endothelium and favors the transmigration from circulation to the intestinal lamina propria. These findings could link the early steps of increased antigen trafficking from the intestinal lumen to the lamina propria with the innate immune response including neutrophil transmigration[21] from the bloodstream to the lamina propria, activation of NK cells and, ultimately, adaptive immune response involving both T and B cell activation.

An upregulation of spliceosome pathways has not been described before in CD. However, our group has previously shown that alternative splicing influences how T regulatory cells suppress the Th17 mediated immune response in active CD subjects [33]. Previous work suggests the microbiome-influenced microenvironment may directly alter Th17 expression [33–35]. Our data shows an upregulation of IL-17. IL-17 can be activated by gliadin, bacteria, and many other factors, and thus it is possible to interpret this finding as implicating the microbiome in the pathogenesis of CD and supports the possibility that early steps leading to loss of tolerance to gluten involve gliadin mistaken as a component of a bacterial pathogen [29, 36].

Villous atrophy, the histological hallmark of CD, has been hypothesized to be related to increased cell destruction/enhanced apoptosis and/or a defect in epithelial cell regeneration. We found an increased expression of CDX genes in active CD subjects compared to remission which could be consistent with induction of stem cell turnover and intestinal regeneration. Additionally, we found an upregulation in molecules related to DNA replication (MCM helicase and Cdc45), confirmed with RT PCR and immunohistochemistry, and of c/myc oncogene, which has been previously shown to be upregulated in CD patients exposed to gluten and associated with CD mucosal damage [26]. Thus, it is possible that the increased apoptosis in active CD [37] is only partially compensated by the accelerated turnover of stem cells and activation of DNA replication, a process that is influenced by the activation of specific oncogenes such as c-myc. This inefficient compensation by the stem cell compartment could explain why some patients with CD, despite strict adherence to the treatment, have persistent enteropathy.

The co-morbidity between CD and other autoimmune diseases is well established [38], however it remains unknown if this association is due to gene clusters increasing the risk of multiple autoimmune diseases, or complication of untreated CD [3, 39]. Vitamin A and its metabolites have a modulatory effect on Th17/Treg balance by improving the differentiation of CD4^+^ cells toward a regulatory phenotype and suppressing the production of Th17 derived proinflammatory cytokines [40]. Several autoimmune diseases characterized by Th17 mediated immune response have been associated with impairments in vitamin A modulatory function [41–43]. We found an upregulation in IL-17 signaling and down-regulation of the vitamin A/retinoic acid pathways in subjects with active CD compared to subjects with CD in remission. We also found evidence to suggest the risk of developing comorbid autoimmune conditions may be particularly high in the active phase of CD as pathways for lupus, T1D, and autoimmune thyroid disease, conditions linked to dysregulation of retinoic acid metabolism, were upregulated [44–46]. Our findings are supported by previous studies suggesting the early diagnosis of CD prevents the development of further autoimmune disease and that comorbid autoimmune conditions improve with CD treatment. [47–52].

**Fig 3:**
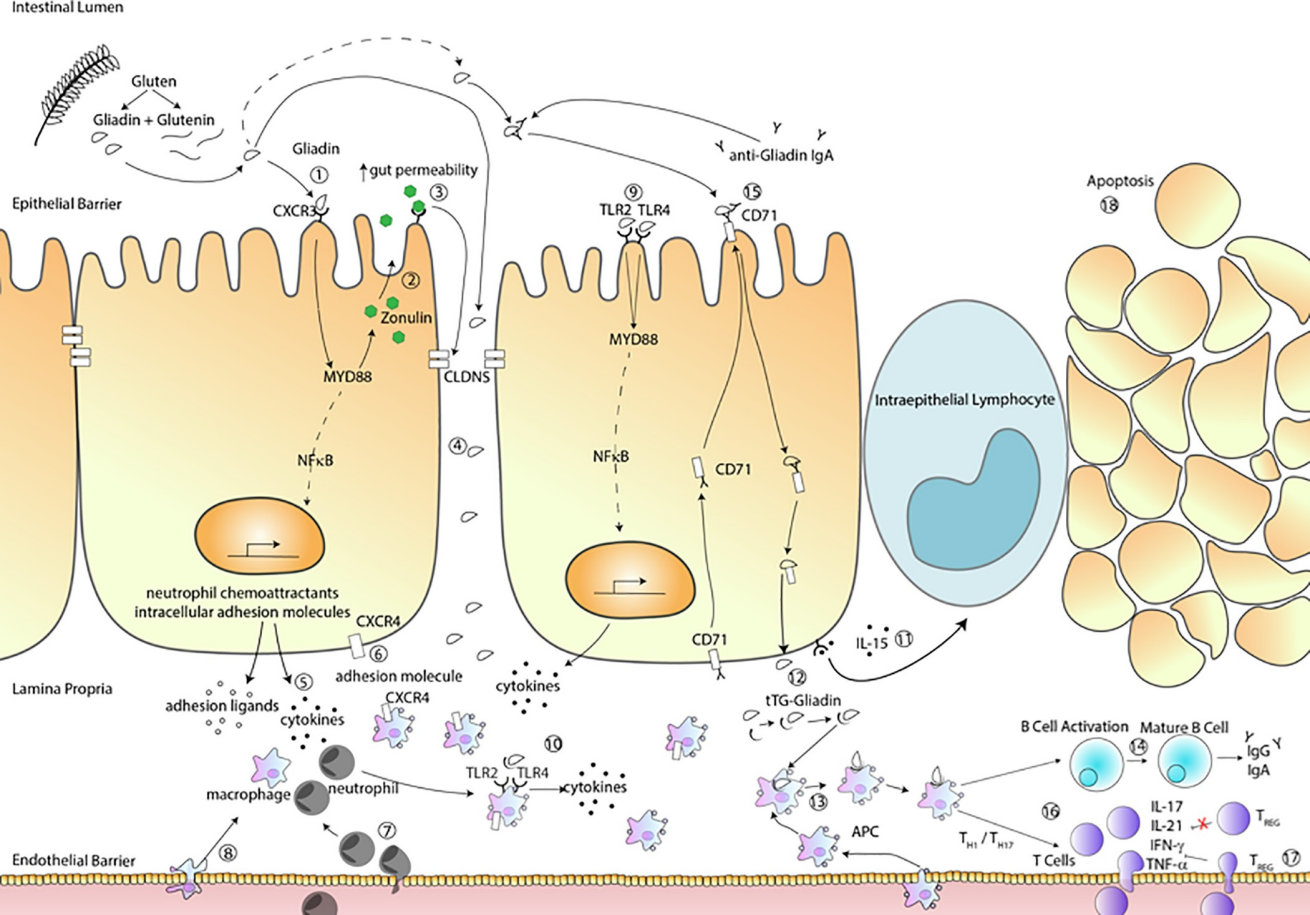
Possible pathogenetic steps leading to CD.

In summary, our study confirmed previous findings based on GWAS and immunological studies pertinent to CD pathogenesis, but also suggests novel areas of investigation related to CD pathogenesis, mucosal repair, and the association with other autoimmune diseases that with further validation may fill knowledge gaps in the chain of events leading to break of tolerance to gluten and onset of CD autoimmunity in genetically-predisposed individuals. Given the large amount of data, we chose to highlight the top three pathways significantly altered in patients with active CD compared to those with remission CD. However, it is possible that differences observed in differentially expressed genes between these groups could be explained by the difference in the crypt depth-villous height ratio since patients with active CD will have shorter villi and therefore may express lower levels of villous genes and elevated expression of crypt genes. The data generated does not localize the altered genes and therefore future validation of these findings is warranted. Additionally, since duodenal biopsies represent a small sample of the intestine, it is possible that some patients found to have CD in remission could have other areas of their intestine with active inflammation which could affect our findings. The power in using RNAseq is that it provides a broad scope of genes and associated pathways involved in the pathogenesis of CD. However, it requires further validation and our validation techniques supported several but not all our findings. Our goal was not to validate all findings but instead provide a roadmap for ourselves and other investigators to investigate new insights into the pathogenesis of CD. Based on these findings, our interpretation of this data combined with previously published work is that gluten exposure followed by increased gut epithelial permeability, coupled with increased endothelial permeability causing the migration of immune cells from the bloodstream to the intestinal lamina propria could represent early events for the initiation of the CD innate immune response (Fig 3). It is possible that this innate immune response to gluten may mimic similar responses triggered by the exposure to pathogens, such as Leishmania (pathway noted to be significantly upregulated in patients with active CD), suggesting that gluten may mistakenly be interpreted as a danger signal by the intestinal mucosal immune surveillance system. Following these early events, cellular insult causing release of intracellular tTG leads to deamidation of gliadin fragments present in the lamina propria following their paracellular passage. Binding of deamidated gliadin to HLA DQ2/8 molecules on the surface of APC and subsequent activation of a Th1/Th17 response, secondary to dysfunctional Tregs due to possible contribution from the spliceosome pathway activation and activation of other adaptive immune cells, could lead to the CD autoimmune enteropathy due to increased apoptosis and defects in the stem cell regenerative compartment (Fig 3).The combination of this information offers novel potential targets for personalized therapies integrative to the gluten free diet and, hopefully, in the future, for possible disease interception (primary prevention) in genetically at-risk subjects. Furthermore, future work using single cell immune profiling to examine patients in the active and remission state of CD, as well as those who fail to respond to the gluten free diet, will be essential to understanding not only CD but other chronic immune based disorders.

**Innate immune response.** Gliadin binding to CXCR3(1) triggers zonulin release(2), changes in intercellular protein complexes gene expression and ultimately, increased gut permeability(3) leading to gliadin fragment passage into the lamina propria(4) (LP). Here, gliadin causes CXCR3-mediated IL-8 secretion(5) that, together with CXCR4/CXCL12 upregulation(6), leads to migration of neutrophils in the LP. This process is facilitated by increased neutrophils’ expression of ITGAM aiding their migration from the bloodstream to the LP(7). B2integrin expression on macrophages and the endothelial ligand ICAM leads to macrophage migration to the LP(8). Gluten also initiates a cascade of anti-microbial responses, including the activation of TLR2/TLR4 with subsequent MyD88-dependent NFkB activation and transcription of pro-inflammatory cytokines(9). Increased expression of TLR2/TLR4 on macrophages leads to the release of anti-microbial molecules(10) secondary to aberration in alternative splicing machinery. The innate immune response is completed by gliadin-dependent IL-15 upregulation, leading to the recruitment intraepithelial lymphocytes which have cytotoxic activity on epithelial cells(11).**Adaptive Immunity** In the LP, gliadin is deamidated and binds to tTG forming a tTG-gliadin complex(12) recognized by antigen presenting cells (APCs) expressing HLA-DQ2/8(13). APC’s presenting gliadin active T helper cells that trigger B cell activation and maturation resulting in production of anti-tTG autoantibodies(14). CD71 translocated to the apical side of intestinal epithelial cells to transport sIgA-gliadin complexes(15). APC also activate TH1 cells releasing proinflammatory cytokines and TH17 cells releasing IL-17 and IL21(16). T regulatory cells migrate from the blood to stop inflammation, however, due to dysfunctional spliceosome pathway, they are not capable to stop inflammation(17). **Ongoing Inflammation** Changes in apoptotic pathways and stem cell turnover-related genes suggest that the celiac enteropathy may be the results of increased apoptosis coupled with incomplete compensation by the stem cell compartment(18).

## Methods

### Study subjects

Seventy-four subjects were approached for inclusion in the study in our clinic or at the time of their clinically indicated endoscopy between June 2014 and August 2015. Of those, 42 subjects met criteria for inclusion into the study. All subjects were 18 years of age or older, carried HLA DQ2 or DQ8, were in good general health and were willing to provide informed consent. Twelve subjects with active CD, 15 subjects with remission CD, and 15 non-CD control subjects with compatible genetics and absence of small intestinal inflammation were included in the study. Characteristics of these patients are shown in Table 2. CD subjects were required to have a diagnosis according to previously described algorithms[53]. In addition to meeting inclusion criteria, subjects with active CD were required to have villous blunting consistent with Marsh 3 on duodenal biopsy and subjects with remission CD must have maintained a gluten free diet for at least one year and have a duodenal biopsy consistent with CD in remission (Marsh 0–2)[54]. Non-CD control subjects were in good health, carried compatible HLA genetics, and were undergoing an endoscopy for evaluation unrelated to CD such as gastroesophageal reflux. Non-CD control subjects were required to have negative celiac serology, normal duodenal biopsies, and no diagnostic abnormalities identified during the clinically indicated endoscopy. Exclusion criteria included receiving systemic immunosuppressive therapy and/or a vaccination within the past 30 days, participation in any clinical research study evaluating an investigational drug or device within the past 30 days, history of other gastrointestinal disease such as inflammatory bowel disease, receipt of anti-coagulant therapy within the previous 30 days, and women who were pregnant or breastfeeding.

**Table 2:** Patient characteristics.

Diagnosis Stratification	Age	Gender	tTG IgA level	DQ2 Homozygous	DQ2 Heterozygous	DQ8	DQ2/DQ8
	(mean)	(%F)	(mean)	(%)	(%)	(%)	(%)
Active CD (N = 12)	36.1	64.3	143.3	33.0	50.0	17.0	0.0
Remission CD (N = 15)^A^	35.5	86.7	12.7	35.7	42.9	21.4	0.0
Non-CD Control (N = 15)	41.8	73.3	1.9	13.3	46.7	20.0	20.0

^A^HLA type of one subject had too low a coverage at DQB1 locus via RNAseq so no allele typing was performed

### Study samples

#### Serology

All subjects underwent venipuncture at the time of the endoscopy procedure. A minimum of 55cc of blood were collected from each participant. Serum was evaluated for antibodies to IgA tissue transglutaminase (antitTG) QUANTA Lite Rh-tTG IgA ELISA (INOVA Diagnostics, San Diego, CA, USA) on the BioFlash platform.

#### HLA determination

Whole blood was collected and stored at -80 degree Celsius. HLA was determined using the DQ-CD Typing Plus (BioDiagne, Palermo, Italy) per the manufacturer’s instructions for all but three subjects included in the analysis. The HLA for the additional three subjects who did not have a whole blood aliquot available for testing were determined by RNA sequencing.

#### Duodenal biopsy

Four duodenal biopsies were obtained during clinically indicated upper endoscopy for each subject. Two biopsies were immediately placed in RNA later and stored at -20^o^ C for RNA sequencing. Two biopsies were placed in 4% paraformaldehyde for four hours at room temperature, washed with 70% ethanol, and stored at room temperature for future use. A histopathologic assessment was completed for all patients by a single pathologist. Results were confirmed by a second pathologist and reported using the modified Marsh scale[54].

#### RNA sequencing

The protocols for RNA extraction and sequencing library preparation were performed as described earlier[55]. Specifically, strand-specific RNA-seq libraries were prepared from 500 ng RNA using KAPA stranded mRNA-Seq Kit (KAPA Biosystems). The libraries were amplified by twelve-cycle PCR. Sequencing was performed on Illumina HiSeq2000 (Illumina) by multiplexed paired-read run with 100 cycles. The resulting FASTQ files were analyzed via FastQC to ensure sufficient data quality.

#### DNAcore RNA extraction and RT-qPCR protocol

For RNA extraction tissue samples were transferred from RNAlater (ThermoFisher, Cat # AM7021) to 1–3 milliliters Qiazol reagent (Qiagen, Cat # 79306). Samples were homogenized on a custom Omni homogenizer (Omni, Cat #SKU: 51-000-1) at 20,000 RPM for 180 seconds. Lysates were phase separated with chloroform and the aqueous phase was purified on the Kingfisher flex (ThermoFisher, Cat # 5400630) with the MagMAX-96 for Microarrays Total RNA Isolation Kit (ThermoFisher, Cat # AM1839) with an additional DNAse (Qiagen, Cat # 79254) step added between the first and second washes. RNA was quantized on the Nanodrop (ThermoFisher, Cat # ND-8000-GL) according to manufactures protocol.

For RT-qPCR, between 37.5 nanograms and 375 nanograms of RNA, per RT-qPCR assay to be run, was mixed with SuperScript VILO Master Mix (ThermoFisher, Cat# 11755500) and cycled according to manufacturer instructions. The cDNA was diluted with nuclease free water to between 0.5 nanograms per microliter and 5 nanograms per microliter. RT-qPCR assays were made by combining water, mastermix (ThermoFisher, Cat #4370074 or Bioline, Cat #CSA-01113), and 20X assay mix. The assay mix is a commercially available mixture of forward and reverse primers combined with a fluorescently labeled and quenched probe sequence (ThermoFisher, Cat# 351372 or LGC BioSearch, Cat# DLO-RFBL-MIX). Samples were run in triplicate on a 384-Well plate (ThermoFisher, Cat# 4343370) by pipetting 5 microliters diluted cDNA and 10 microliters of appropriate RT-PCR assay into each well. The 384-well plate was covered with an optically clear seal (Agilent, Cat# 16985–001), spun down, and read on an ABI 7900HT Fast Real-Time PCR System with 384-Well Block Module and Automation Accessory (ThermoFisher, Cat# 4329002) for 40 cycles according to the specifics of the mastermix used.

#### Immunohistochemistry

Immunohistochemistry of paraffin embedded duodenal biopsies were performed on well oriented 5μm thick paraffin embedded samples from human duodenum of patients. Two samples from patients diagnosed with acute CD (Marsh 3a-c), patients with CD in remission (Marsh 0–1), and normal mucosa subjects (Marsh 0), were obtained from the Pathology Clinics at MGH for this purpose. Duodenal sections from control donors, donors with active celiac disease (CDA) or remission celiac disease (CDR) were stained for IHC using Vector Laboratories Vectastain Elite Kit and Vector DAB Kit per manufacturer’s instructions. Briefly, sections were deparaffinized and hydrated using xylenes and a graded ethanol series. Antigen retrieval was performed using sodium citrate buffer at pH 6.0. Sections were blocked against endogenous peroxidase activity using 0.3% hydrogen peroxide in water for thirty minutes. After washing and blocking using diluted normal serum (Vector Laboratories), primary antibodies against MCM2 (Thermo Fisher clone 2BE, Cat #MA5-15893) and CDC45 (Sigma Prestige Antibodies, Cat# HPA00614) were used at a concentration of 1:400 and 1:150, respectively, overnight at 4 degrees. The next day, slides were washed and treated with diluted biotinylated secondary antibodies (Vector Laboratories) for 30 minutes at room temperature. Sections were then washed and incubated with Vectastain Elite ABC reagent followed by peroxidase substrate solution (Vector DAB kit) until developed. Sections were mounted and imaged using an inverted light microscope (Nikon Eclipse TE2000-5) at 10x magnification. Post-acquisition analysis was conducted using ImageJ [56] and the IHC toolbox plugin[57].

### Statistical analysis

#### Derivation of differentially expressed gene signatures

The transcriptome sequencing reads were mapped to the hg19 genome reference using the OmicSoft ArrayStudio RNASeq analysis pipeline (Qiagen Inc) with two mismatches allowed. The resulting read counts summarized at gene level represented the raw gene expression measures. We normalized the raw gene expression in each sample by global scaling to match the median library size (i.e. the total number of mapped reads) as well as the upper quantile of the gene-level read counts across all samples, as described in previous studies[58]. An empirical minimum read count of 10 was applied to flag the “absence” and “presence” of genes in each sample. For each comparison between two groups of samples, we first eliminated genes that were not flagged as “presence” in at least 60% of the samples of the higher expressing group. Next, fold changes associated with the comparison were calculated as the ratio between the arithmetic mean expressions in the two groups. The statistical significance (p-value) of the differential expressions was assessed under negative binomial distribution models using DESeq package version 1.6[59]. At the end, we selected genes with fold changes no less than 1.5 in either up or down directions with a p-values of at least 0.05 as the significantly perturbed gene signatures.

#### Gene set enrichment analysis(GSEA) and pathway analysis

In the active CD versus remission CD comparison we performed the Gene Set Enrichment Analysis (GSEA). The input contained all gene symbols detectable in the RNA sequencing, together with their fold changes between the active and remission CD subjects to provide the needed rank information in the GSEA. The fold changes smaller than 1 were converted to the negative reciprocal values. The analysis was performed using the build-in “GSEA” function in the R package “clusterProfiler”[60]. The gene sets used in the analysis were the curated KEGG canonical pathways in the MSigDB (version: c2.cp.kegg.v6.1.symbols.gmt). The pathways with adjusted p-value < = 0.05 were reported. To analyze the enriched pathways in the unique signatures to the active CD, and in the common signatures to the active and remission CD, we executed the Pathway Enrichment module of NextBio, which applies a running Fisher algorithm over the MSigDB curated canonical pathways[16]. Note that GSEA is not applicable here as the signatures were subjected to the differential gene expression selection criteria as well as Venn Diagram analysis (Fig 2), and thus no longer contain the genome-wide genes. Pathways with a running Fisher test p-value < = 0.001 and at least 3 genes occurring in the signatures were considered significant and reported.

#### Study approval

Written informed consent was obtained from subjects prior to inclusion in the study according to the standards outlined and approved by the Partners Human Resource Committee Institutional Review Board.

## Supporting information

S1 Table Pathways unique to patients with active CD. (DOCX)

S1 Fig Gene Expression Signatures Unique to Active CD. Heatmap shows non-CD control subjects (blue), active CD subjects (green) and remission CD subjects (pink) with each subject’s data represented vertically. The color scale show gene expression with dark blue indicating downregulation, light blue indicating decreased expression, light red indicating increased expression, and red indicating upregulation.
(TIF)

S2 Fig Gene Expression Common to Active and Remission CD. Heatmap shows non-CD control subjects (blue), active CD subjects (green) and remission CD subjects (pink) with each subject’s data represented vertically. The color scale show gene expression with dark blue indicating downregulation, light blue indicating decreased expression, light red indicating increased expression, and red indicating upregulation.
(TIF)

S3 Fig RTPCR Results in a subset of genes in healthy control patients, patients with active CD, and patients with CD in remission. (TIF)

S4 Fig Immunohistochemistry results in a subset of healthy control patients, patients with active CD, and patients with CD in remission. (TIF)

S5 Fig KEGG pathway cytokine-cytokine receptor interaction. We performed a subtraction analysis using GSEA for patients with active CD compared to those with CD in remission. Genes identified as significant are highlighted in red against a background of other known genes in the pathway.
(TIF)

S6 Fig KEGG pathway chemokine signaling pathway. We performed a subtraction analysis using GSEA for patients with active CD compared to those with CD in remission. Genes identified as significant are highlighted in red against a background of other known genes in the pathway.
(TIF)

S7 Fig KEGG pathway cell cycle. We performed a subtraction analysis using GSEA for patients with active CD compared to those with CD in remission. Genes identified as significant are highlighted in red against a background of other known genes in the pathway.
(TIF)

S8 Fig KEGG pathway cell adhesion molecules. We performed a subtraction analysis using GSEA for patients with active CD compared to those with CD in remission. Genes identified as significant are highlighted in red against a background of other known genes in the pathway.
(TIF)

S9 Fig KEGG pathway spliceosome. We performed a subtraction analysis using GSEA for patients with active CD compared to those with CD in remission. Genes identified as significant are highlighted in red against a background of other known genes in the pathway.
(TIF)

S10 Fig KEGG pathway retinol metabolism. We performed a subtraction analysis using GSEA for patients with active CD compared to those with CD in remission. Genes identified as significant are highlighted in red against a background of other known genes in the pathway.
(TIF)

S11 Fig KEGG pathway systemic lupus erythematosus. We performed a subtraction analysis using GSEA for patients with active CD compared to those with CD in remission. Genes identified as significant are highlighted in red against a background of other known genes in the pathway.
(TIF)

S12 Fig KEGG pathway type 1 diabetes mellitus. We performed a subtraction analysis using GSEA for patients with active CD compared to those with CD in remission. Genes identified as significant are highlighted in red against a background of other known genes in the pathway.
(TIF)

S13 Fig KEGG pathway autoimmune thyroid disease. We performed a subtraction analysis using GSEA for patients with active CD compared to those with CD in remission. Genes identified as significant are highlighted in red against a background of other known genes in the pathway.
(TIF)

## Abbreviations

CD: Celiac Disease
CLDN: Claudin
EGF: Epidermal Growth Factor
GPCR: G protein coupled receptor
GSEA: Gene Set Enrichment Analysis
HLA: Human Leukocyte Antigen
IEL: Intraepithelial Lymphocyte
ICAMs: Intercellular Adhesion Molecules
TCR: T cell receptor
anti-tTG2: Tissue Transglutaminase
T1D: Type 1 diabetes
