# Supplementary material for: RNA sequencing of intestinal mucosa reveals novel pathways functionally linked to celiac disease pathogenesis
Source: PLoS One. 2019 Apr 18;14(4):e0215132. doi: 10.1371/journal.pone.0215132 (PMC6472737; doi:10.1371/journal.pone.0215132)
Supplement: S1 Table — (DOCX) [file pone.0215132.s001.docx]

S1 Table: Pathways Unique to Active CD

| **Biogroup name** | **#genes** | **direction** | **p-value** |
| --- | --- | --- | --- |
| Genes involved in Cell Cycle, Mitotic | 37 | up | 5.30E-41 |
| Genes involved in Cell Cycle | 39 | up | 1.00E-39 |
| Genes involved in DNA Replication | 26 | up | 7.90E-31 |
| Genes involved in Mitotic M-M/G1 phases | 23 | up | 7.50E-28 |
| PLK1 signaling events | 16 | up | 3.00E-27 |
| Genes involved in Mitotic Prometaphase | 18 | up | 1.30E-25 |
| FOXM1 transcription factor network | 12 | up | 1.80E-20 |
| Retinol metabolism | 12 | down | 6.20E-19 |
| Drug metabolism - other enzymes | 10 | down | 6.20E-18 |
| Cell cycle | 15 | up | 6.40E-18 |
| Starch and sucrose metabolism | 10 | down | 7.70E-18 |
| Steroid hormone biosynthesis | 10 | down | 1.40E-17 |
| Ascorbate and aldarate metabolism | 8 | down | 1.70E-16 |
| Metabolism of xenobiotics by cytochrome P450 | 11 | down | 1.90E-16 |
| Drug metabolism - cytochrome P450 | 11 | down | 2.50E-16 |
| Aurora B signaling | 10 | up | 3.60E-16 |
| Pentose and glucuronate interconversions | 8 | down | 5.00E-16 |
| Genes involved in Glucuronidation | 7 | down | 2.80E-15 |
| Genes involved in Kinesins | 8 | up | 2.80E-15 |
| Porphyrin and chlorophyll metabolism | 8 | down | 1.50E-14 |
| Genes involved in Cyclin A/B1 associated events during G2/M transition | 7 | up | 3.60E-14 |
| Genes involved in Biological oxidations | 11 | down | 1.90E-13 |
| Genes involved in Mitotic G2-G2/M phases | 9 | up | 2.10E-11 |
| Genes involved in Mitotic G1-G1/S phases | 10 | up | 3.10E-11 |
| Genes involved in Phase II conjugation | 7 | down | 8.90E-11 |
| Progesterone-mediated oocyte maturation | 9 | up | 9.50E-11 |
| E2F transcription factor network | 8 | up | 2.40E-10 |
| Genes involved in Interferon gamma signaling | 7 | up | 2.40E-10 |
| Cytokine-cytokine receptor interaction | 10 | up | 3.40E-10 |
| Genes involved in Hemostasis | 13 | up | 9.80E-10 |
| Leishmania infection | 5 | up | 1.10E-09 |
| Oocyte meiosis | 9 | up | 1.20E-09 |
| Genes involved in Cell Cycle Checkpoints | 9 | up | 1.70E-09 |
| Genes involved in Lipid digestion, mobilization, and transport | 6 | down | 1.80E-09 |
| Genes involved in Regulation of mitotic cell cycle | 8 | up | 1.90E-09 |
| Aurora A signaling | 6 | up | 2.20E-09 |
| Genes involved in Factors involved in megakaryocyte development and platelet production | 8 | up | 4.90E-09 |
| Genes involved in G1/S Transition | 8 | up | 5.80E-09 |
| Genes involved in G2/M Checkpoints | 6 | up | 8.00E-09 |
| Genes involved in MHC class II antigen presentation | 7 | up | 9.70E-09 |
| Genes involved in Cytokine Signaling in Immune system | 11 | up | 1.30E-08 |
| p73 transcription factor network | 7 | up | 1.30E-08 |
| Role of Ran in mitotic spindle regulation | 4 | up | 5.10E-08 |
| Genes involved in Transmembrane transport of small molecules | 11 | down | 5.40E-08 |
| FOXA2 and FOXA3 transcription factor networks | 5 | down | 6.30E-08 |
| Calcineurin-regulated NFAT-dependent transcription in lymphocytes | 5 | up | 6.40E-08 |
| Genes involved in E2F mediated regulation of DNA replication | 5 | up | 1.50E-07 |
| p53 signaling pathway | 6 | up | 1.50E-07 |
| Genes involved in Interferon Signaling | 8 | up | 1.70E-07 |
| Genes involved in Lipoprotein metabolism | 4 | down | 2.40E-07 |
| IL23-mediated signaling events | 4 | up | 2.60E-07 |
| Genes involved in Metabolism of lipids and lipoproteins | 10 | down | 3.10E-07 |
| Genes involved in Chemokine receptors bind chemokines | 4 | up | 3.40E-07 |
| CEN complex | 5 | up | 4.60E-07 |
| Genes involved in G1/S-Specific Transcription | 4 | up | 5.40E-07 |
| IL12-mediated signaling events | 4 | up | 1.30E-06 |
| Cell Cycle: G2/M Checkpoint | 4 | up | 1.50E-06 |
| Chemokine signaling pathway | 5 | up | 1.70E-06 |
| Genes involved in G0 and Early G1 | 4 | up | 1.70E-06 |

| Allograft rejection | 3 | up | 1.90E-06 |
| --- | --- | --- | --- |
| Systemic lupus erythematosus | 4 | up | 2.00E-06 |
| Genes involved in APC-Cdc20 mediated degradation of Nek2A | 4 | up | 3.40E-06 |
| Genes involved in Chylomicron-mediated lipid transport | 3 | down | 3.70E-06 |
| Genes involved in Recruitment of mitotic centrosome proteins and complexes | 5 | up | 4.70E-06 |
| Genes involved in E2F-enabled inhibition of pre-replication complex formation | 3 | up | 5.00E-06 |
| ATF-2 transcription factor network | 4 | up | 6.80E-06 |
| Activation of Src by Protein-tyrosine phosphatase alpha | 3 | up | 6.80E-06 |
| Sonic Hedgehog (SHH) Receptor Ptc1 Regulates cell cycle | 3 | up | 6.80E-06 |
| Genes involved in Deposition of New CENPA-containing Nucleosomes at the Centromere | 5 | up | 7.30E-06 |
| Homologous recombination | 4 | up | 7.70E-06 |
| Genes involved in S Phase | 6 | up | 9.60E-06 |
| Genes involved in APC/C:Cdc20 mediated degradation of mitotic proteins | 5 | up | 1.10E-05 |
| RC complex during G2/M-phase of cell cycle | 3 | up | 1.20E-05 |
| Genes involved in Peptide ligand-binding receptors | 5 | up | 1.30E-05 |
| Glycolysis / Gluconeogenesis | 4 | down | 1.30E-05 |
| Genes involved in G alpha (i) signalling events | 6 | up | 1.40E-05 |
| Genes involved in Unwinding of DNA | 3 | up | 1.40E-05 |
| Genes involved in Phase 1 - Functionalization of compounds | 4 | down | 1.60E-05 |
| Genes involved in Metabolism of carbohydrates | 6 | down | 2.00E-05 |
| Autoimmune thyroid disease | 4 | up | 2.00E-05 |
| Genes involved in Interferon alpha/beta signaling | 4 | up | 2.20E-05 |
| Genes involved in Formation of Fibrin Clot (Clotting Cascade) | 3 | down | 2.60E-05 |
| Natural killer cell mediated cytotoxicity | 3 | up | 2.90E-05 |
| Regulation of nuclear SMAD2/3 signaling | 3 | up | 3.20E-05 |
| Downstream signaling in CD8+ T cells | 3 | up | 3.40E-05 |
| IL2 signaling events mediated by STAT5 | 3 | up | 3.90E-05 |
| Genes involved in Phosphorylation of the APC/C | 3 | up | 4.60E-05 |
| Genes involved in Activation of the pre-replicative complex | 3 | up | 5.10E-05 |
| Genes involved in Class A/1 (Rhodopsin-like receptors) | 7 | up | 5.30E-05 |
| Genes involved in Synthesis of DNA | 5 | up | 6.00E-05 |
| Genes involved in Loss of Nlp from mitotic centrosomes | 4 | up | 6.80E-05 |
| Genes involved in SLC-mediated transmembrane transport | 6 | down | 7.00E-05 |
| Genes involved in APC/C:Cdc20 mediated degradation of Cyclin B | 3 | up | 7.10E-05 |
| Genes involved in Activation of ATR in response to replication stress | 3 | up | 8.00E-05 |
| Genes involved in Inhibition of the proteolytic activity of APC/C required for the onset of anaphase by mitotic spindle checkpoint components | 3 | up | 8.90E-05 |
| ATR signaling pathway | 3 | up | 0.0001 |
| Genes involved in GPCR ligand binding | 7 | up | 0.0001 |
| Genes involved in ABC-family proteins mediated transport | 3 | down | 0.0001 |
| Genes involved in Chromosome Maintenance | 5 | up | 0.0002 |
| SHP2 signaling | 3 | up | 0.0002 |
| Regulation of Telomerase | 3 | up | 0.0002 |
| T cell receptor signaling pathway | 3 | up | 0.0002 |
| IL2-mediated signaling events | 3 | up | 0.0002 |
| Complement and coagulation cascades | 3 | down | 0.0003 |
| Genes involved in G alpha (s) signalling events | 4 | down | 0.0003 |
| Jak-STAT signaling pathway | 5 | up | 0.0003 |
| IL4-mediated signaling events | 3 | up | 0.0003 |
| Genes involved in DNA strand elongation | 3 | up | 0.0003 |
| Fanconi anemia pathway | 3 | up | 0.0004 |
| Genes involved in Cytochrome P450 - arranged by substrate type | 3 | down | 0.0005 |
| Genes involved in Glucose metabolism | 3 | down | 0.0005 |
| Genes involved in Interleukin-2 signaling | 3 | up | 0.0005 |
| PPAR signaling pathway | 3 | down | 0.0005 |
| Endothelins | 3 | down | 0.0006 |
| Genes involved in Signaling by Interleukins | 4 | up | 0.0007 |
| Genes involved in M/G1 Transition | 3 | up | 0.0009 |
| Hematopoietic cell lineage | 3 | up | 0.0009 |
| AP-1 transcription factor network | 3 | up | 0.001 |
| HIF-1-alpha transcription factor network | 3 | down | 0.001 |
