# Supplementary figures and images for: RNA sequencing of intestinal mucosa reveals novel pathways functionally linked to celiac disease pathogenesis
Source: PLoS One. 2019 Apr 18;14(4):e0215132. doi: 10.1371/journal.pone.0215132 (PMC6472737; doi:10.1371/journal.pone.0215132)

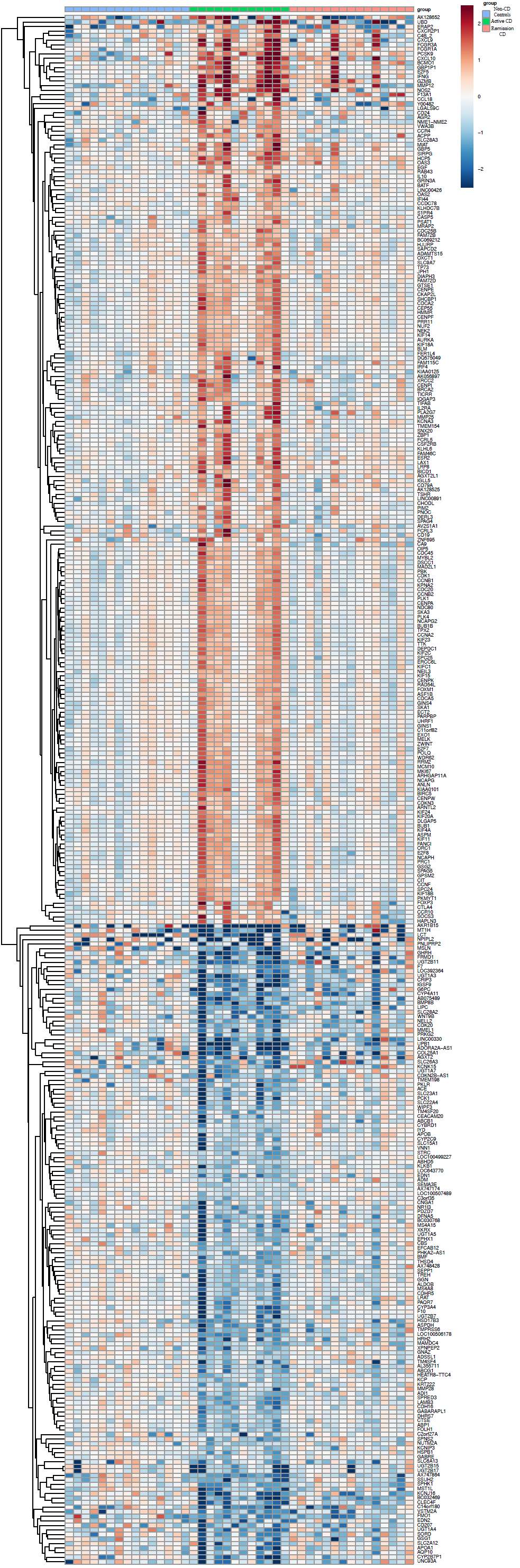

Supplement: S1 Fig — Heatmap shows non-CD control subjects (blue), active CD subjects (green) and remission CD subjects (pink) with each subject’s data represented vertically. The color scale show gene expression with dark blue indicating downregulation, light blue indicating decreased expression, light red indicating increased expression, and red indicating upregulation. (TIF) [file pone.0215132.s002.tif]

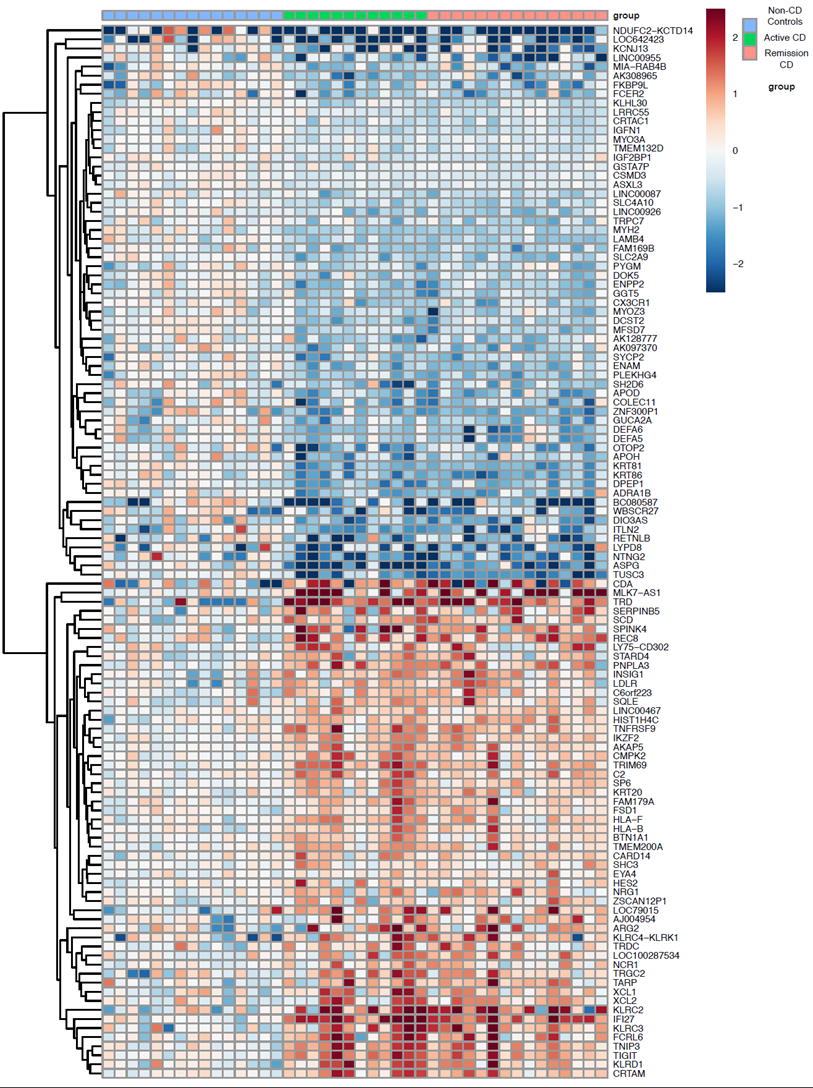

Supplement: S2 Fig — Heatmap shows non-CD control subjects (blue), active CD subjects (green) and remission CD subjects (pink) with each subject’s data represented vertically. The color scale show gene expression with dark blue indicating downregulation, light blue indicating decreased expression, light red indicating increased expression, and red indicating upregulation. (TIF) [file pone.0215132.s003.tif]

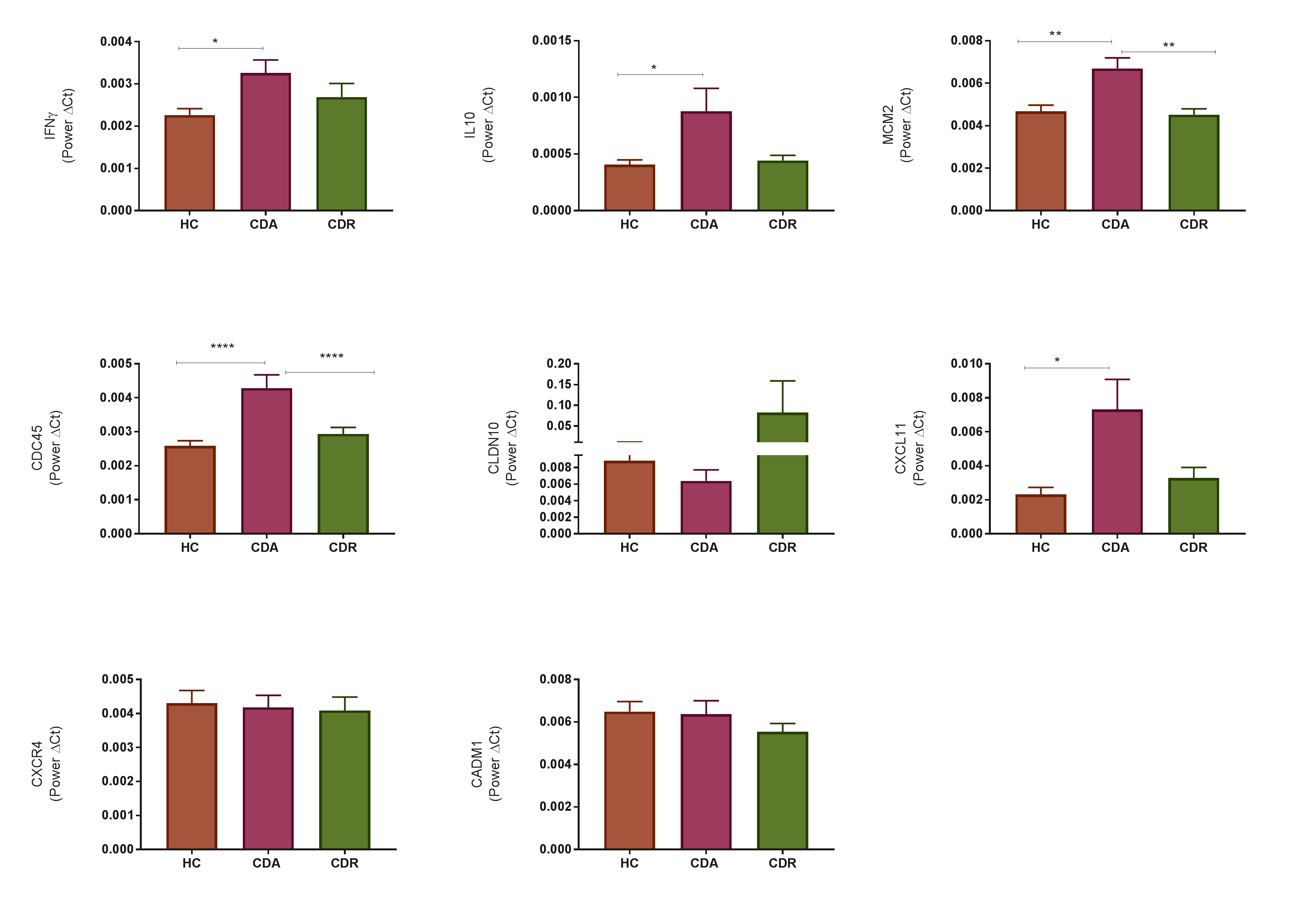

Supplement: S3 Fig — (TIF) [file pone.0215132.s004.tif]

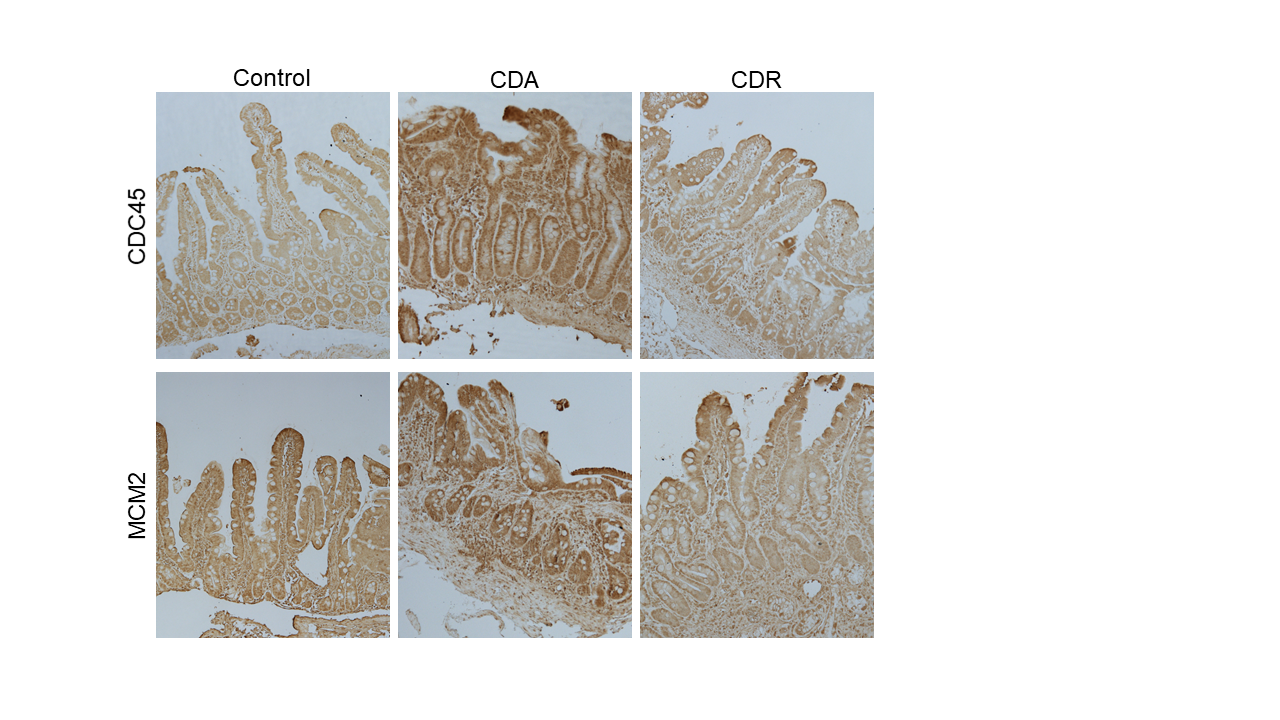

Supplement: S4 Fig — (TIF) [file pone.0215132.s005.TIF]

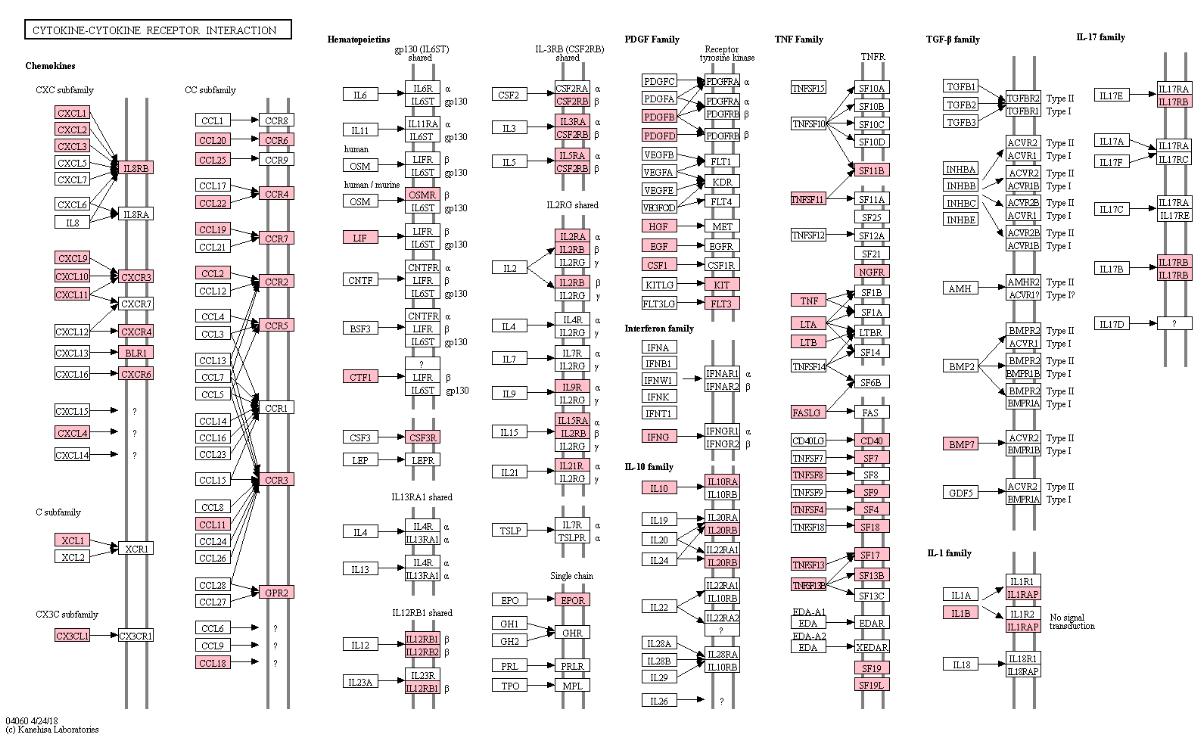

Supplement: S5 Fig — Genes identified as significant are highlighted in red against a background of other known genes in the pathway. (TIF) [file pone.0215132.s006.tif]

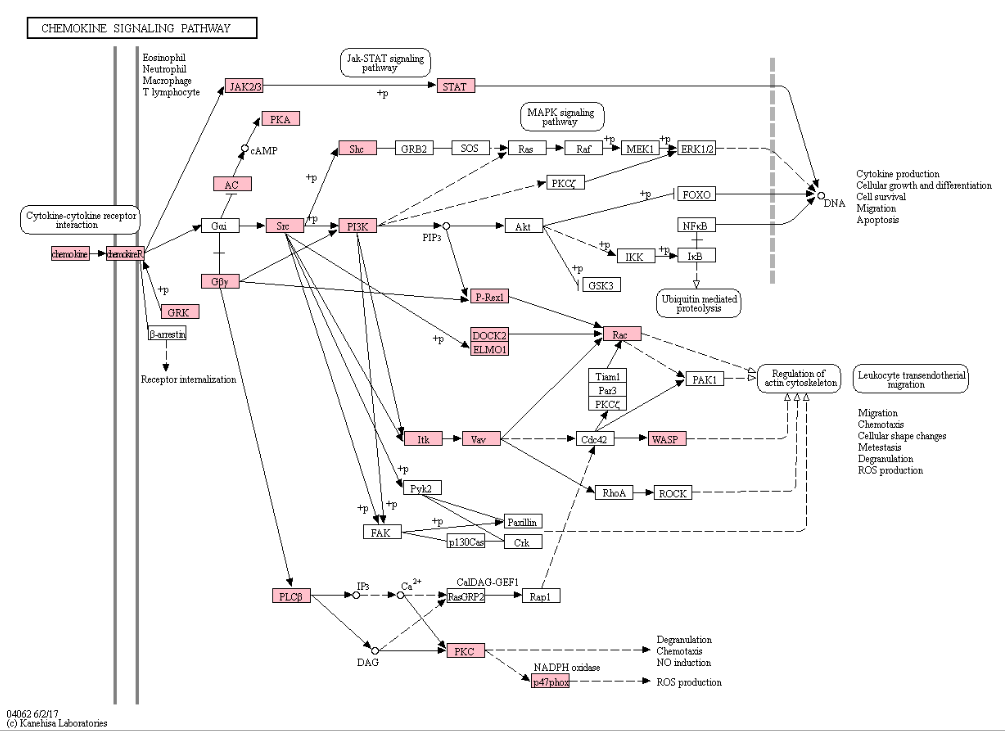

Supplement: S6 Fig — We performed a subtraction analysis using GSEA for patients with active CD compared to those with CD in remission. Genes identified as significant are highlighted in red against a background of other known genes in the pathway. (TIF) [file pone.0215132.s007.tif]

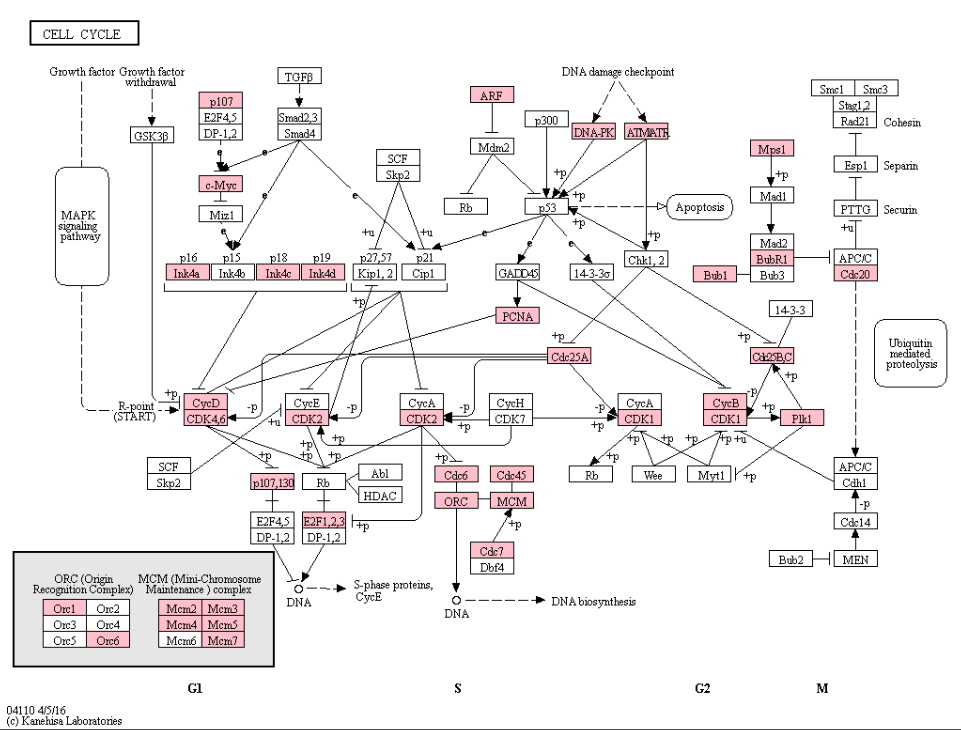

Supplement: S7 Fig — We performed a subtraction analysis using GSEA for patients with active CD compared to those with CD in remission. Genes identified as significant are highlighted in red against a background of other known genes in the pathway. (TIF) [file pone.0215132.s008.tif]

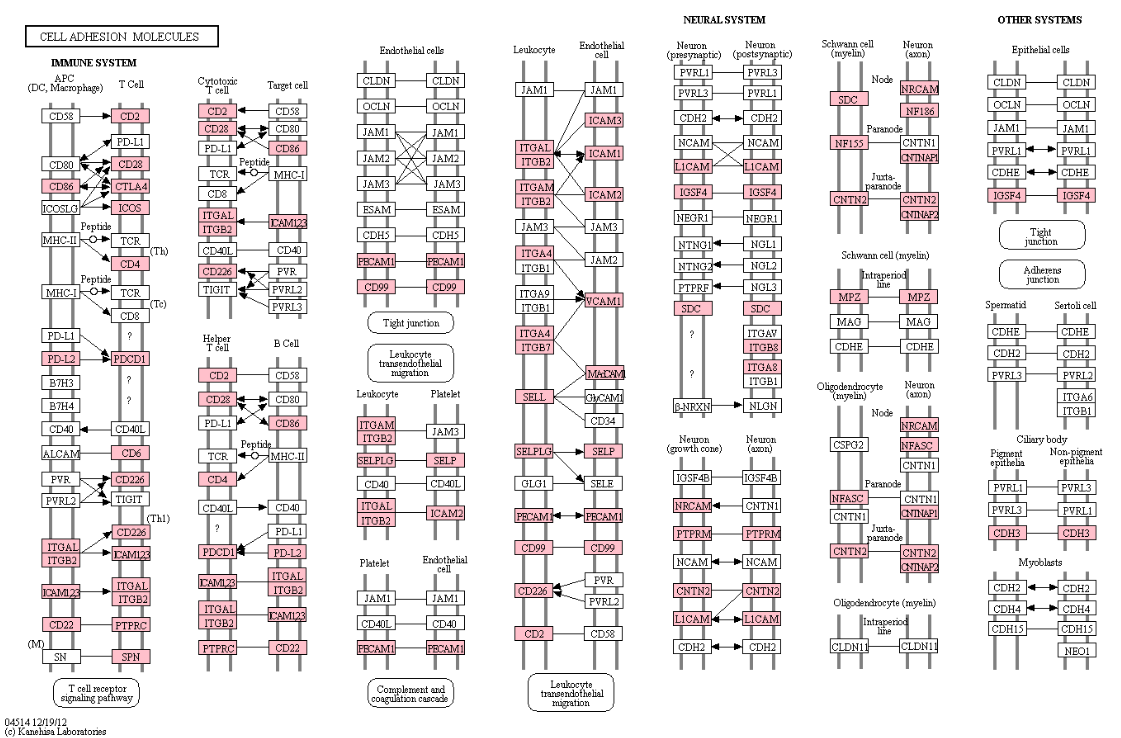

Supplement: S8 Fig — We performed a subtraction analysis using GSEA for patients with active CD compared to those with CD in remission. Genes identified as significant are highlighted in red against a background of other known genes in the pathway. (TIF) [file pone.0215132.s009.tif]

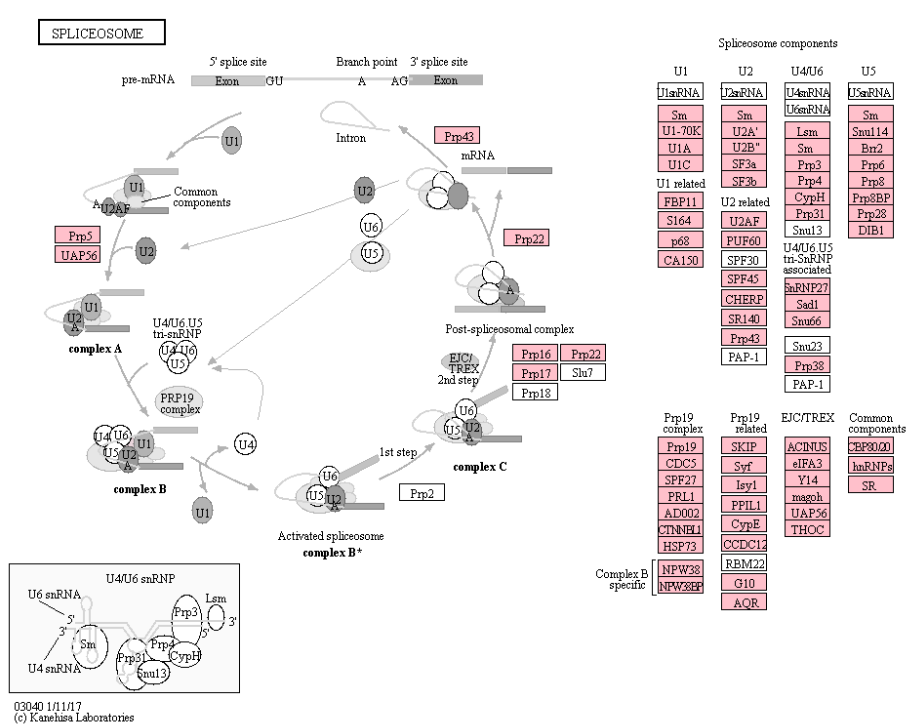

Supplement: S9 Fig — We performed a subtraction analysis using GSEA for patients with active CD compared to those with CD in remission. Genes identified as significant are highlighted in red against a background of other known genes in the pathway. (TIF) [file pone.0215132.s010.tif]

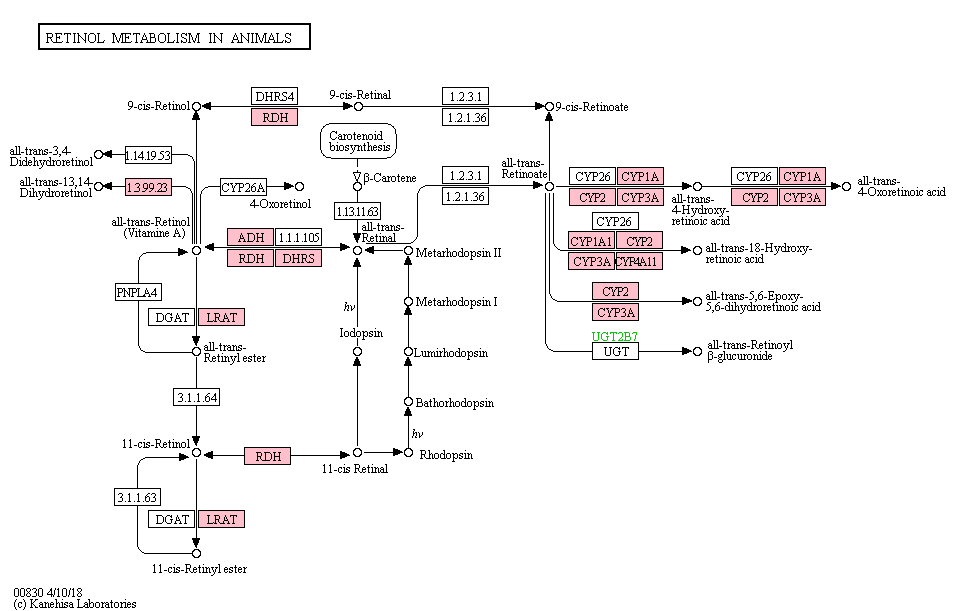

Supplement: S10 Fig — We performed a subtraction analysis using GSEA for patients with active CD compared to those with CD in remission. Genes identified as significant are highlighted in red against a background of other known genes in the pathway. (TIF) [file pone.0215132.s011.tif]

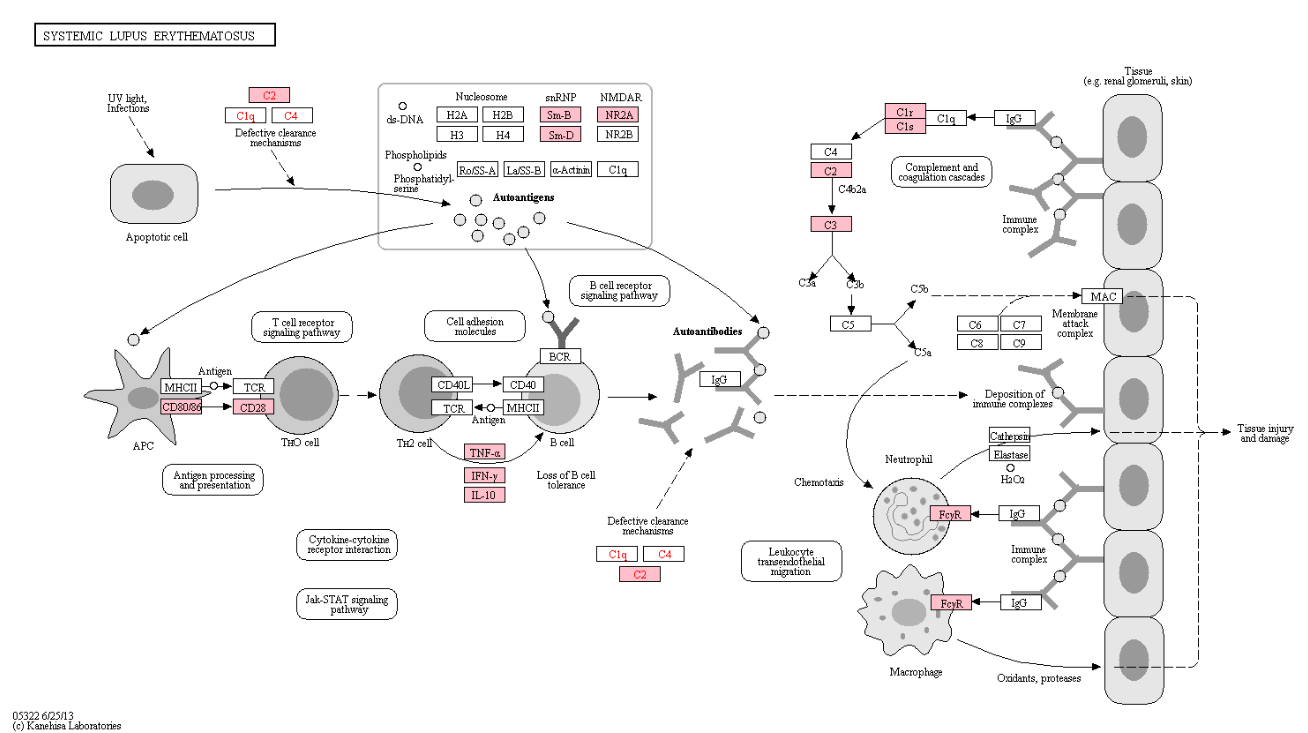

Supplement: S11 Fig — We performed a subtraction analysis using GSEA for patients with active CD compared to those with CD in remission. Genes identified as significant are highlighted in red against a background of other known genes in the pathway. (TIF) [file pone.0215132.s012.tif]

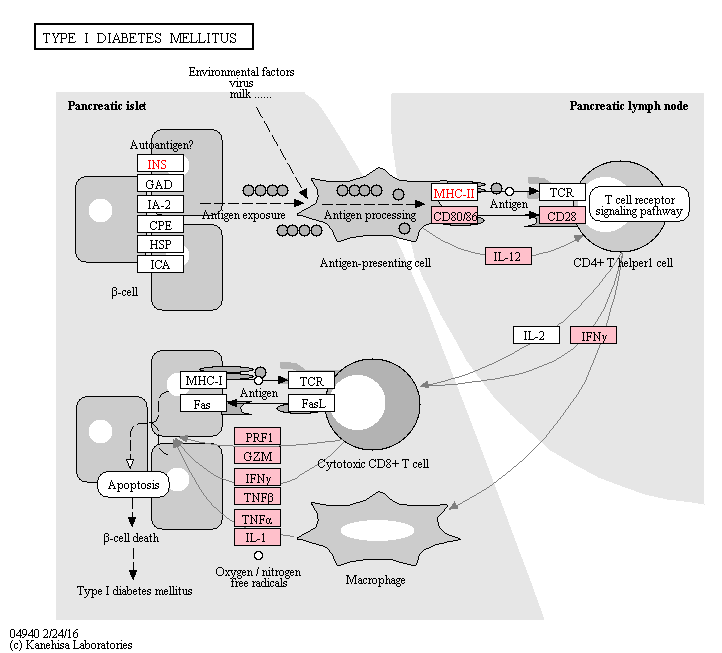

Supplement: S12 Fig — We performed a subtraction analysis using GSEA for patients with active CD compared to those with CD in remission. Genes identified as significant are highlighted in red against a background of other known genes in the pathway. (TIF) [file pone.0215132.s013.tif]

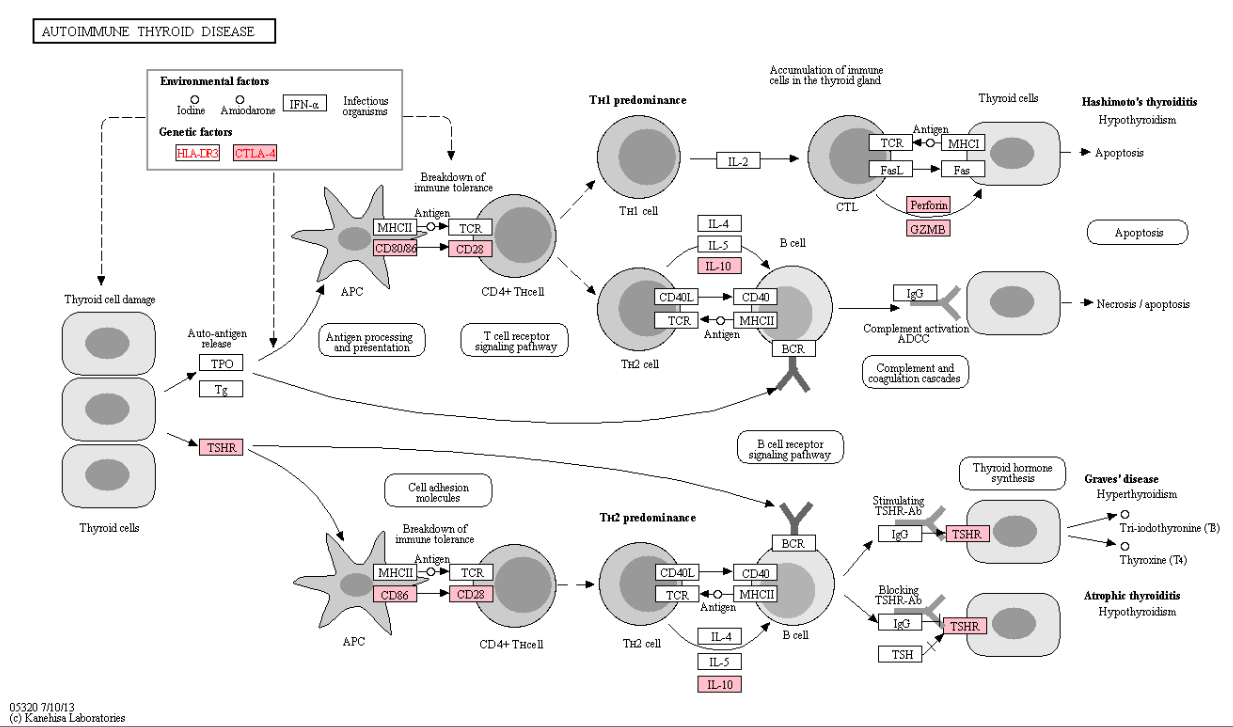

Supplement: S13 Fig — We performed a subtraction analysis using GSEA for patients with active CD compared to those with CD in remission. Genes identified as significant are highlighted in red against a background of other known genes in the pathway. (TIF) [file pone.0215132.s014.tif]
